# Comprehensive Chemical Profiling and Multidirectional Biological Investigation of Two Wild *Anthemis* Species (*Anthemis tinctoria* var. *Pallida* and *A. cretica* subsp. *tenuiloba)*: Focus on Neuroprotective Effects

**DOI:** 10.3390/molecules24142582

**Published:** 2019-07-16

**Authors:** Giustino Orlando, Gokhan Zengin, Claudio Ferrante, Maurizio Ronci, Lucia Recinella, Ismail Senkardes, Reneta Gevrenova, Dimitrina Zheleva-Dimitrova, Annalisa Chiavaroli, Sheila Leone, Simonetta Di Simone, Luigi Brunetti, Carene Marie Nancy Picot-Allain, Mohamad Fawzi Mahomoodally, Kouadio Ibrahime Sinan, Luigi Menghini

**Affiliations:** 1 Department of Pharmacy, University “G. d’Annunzio” of Chieti-Pescara, 66100 Chieti, Italy; 2 Department of Biology, Faculty of Science, Selcuk University, Konya 42130, Turkey; 3 Department of Medical, Oral and Biotechnological Sciences, University “G. d’Annunzio” of Chieti-Pescara, 66100 Chieti, Italy; 4 Department of Pharmaceutical Botany, Faculty of Pharmacy, Marmara University, Istanbul 34668, Turkey; 5 Department of Pharmacognosy, Faculty of Pharmacy, Medical University of Sofia, 1431 Sofia, Bulgaria; 6 Department of Health Sciences, Faculty of Science, University of Mauritius, Réduit 80837, Mauritius

**Keywords:** *Anthemis*, oxidative stress, neurotransmission, proteomic, phytomedicine

## Abstract

Ethyl acetate (EA), methanol (MeOH), and aqueous extracts of aerial parts of *Anthemis tinctoria* var. *pallida* (ATP) and *A. cretica* subsp. *tenuiloba* (ACT) were investigated for their phenol and flavonoid content, antioxidant, and key enzyme inhibitory potentials. All extracts displayed antiradical effects, with MeOH and aqueous extracts being a superior source of antioxidants. On the other hand, EA and MeOH extracts were potent against AChE and BChE. Enzyme inhibitory effects against tyrosinase and α-glucosidase were observed, as well. We also studied *Anthemis* extracts in an ex vivo experimental neurotoxicity paradigm. We assayed extract influence on oxidative stress and neurotransmission biomarkers, including lactate dehydrogenase (LDH) and serotonin (5-HT), in isolated rat cortex challenged with K^+^ 60 mM Krebs-Ringer buffer (excitotoxicity stimulus). An untargeted proteomic analysis was finally performed in order to explore the putative mechanism in the brain. The pharmacological study highlighted the capability of ACT water extract to blunt K^+^ 60 mM increase in LDH level and 5-HT turnover, and restore physiological activity of specific proteins involved in neuron morphology and neurotransmission, including NEFMs, VAMP-2, and PKCγ, thus further supporting the neuroprotective role of ACT water extract.

## 1. Introduction

*Anthemis* L is the second largest genus in Asteraceae family including more than 210 species, which are distributed in western Eurasia, Mediterranean and a small part of eastern Africa. According to the Flora of Turkey, the Anthemideae are divided into three subgenera (*Anthemis, Maruta* and *Cota*) and the subgenus *Anthemis* includes four sections; *Hiorthia*, *Anthemis*, *Maruta*, and *Chia* [1,2,3]. In Turkey, the genus is represented by 81 taxa belonging to 51 species, 29 (54%) of which are endemic. Species belonging to *Anthemis* genus are commonly referred to as “Papatya”, in Turkey [1,4,5].

The species belonging to *Anthemis* genus are known to possess various biological properties and have found broad use in pharmaceutics, cosmetics, and food chemistry. The flowers of *Anthemis* species are well-documented for their use as antiseptic and healing herbs, with flavonoids, and essential oils being the main active components [2,6]. Extracts, tinctures, salves, and tisanes are extensively used as antispasmodic, anti-inflammatory, antibacterial and sedative agents, in Europe [5]. Extracts are also used to clean wounds and ulcers, and as therapy for irradiated skin injuries, cystitis and dental afflictions [2]. The antimicrobial activity of essential oils of several *Anthemis* species have been previously reported [7,8,9,10]. Moreover, *Anthemis* species are widely used to treat intestinal disorders, kidney stones, and hemorrhoids in traditional medicine. The plant is also used as antispasmodic medications and to stimulate menstrual flow. It is documented that the seed oil has been used in the treatment of earaches and deafness [11,12,13].

*Anthemis* genus is mainly characterized by the presence of sesquiterpene lactones, flavonoids and essential oils. Sesquiterpene lactones belonging to germacranolides, eudesmanolides, and guaianolides have been gained attention because of their chemo-ecological functions, biological activities and taxonomic significance. They are the major classes of secondary metabolites in *Anthemis* genus [2,3,14]. The essential oil compositions of several *Anthemis* species has also been investigated [2,12,15,16].

*A. tinctoria* var. *pallida* (ATP) is a rounded perennial plant measuring between 20 to 45 cm. The flowers are white or cream [17]. ATP, commonly known as yellow chamomile, produces a yellow dye used in food industry for production of diary and butchery products. Decoction of ATP flower is traditionally taken to treat shortness of breath, bronchitis, stomachache, anxiety, and to strengthen hair [18]. Aerial part of *Anthemis* species has been reported to exhibit antimicrobial property [17]. However, there is no record of the use of *A. cretica* subsp. *tenuiloba* (ACT) by folk populations, in Turkey.

To the best of our knowledge, there are no reports in literature investigating chemical profile and biological activities of ATP and ACT. Thus, we aimed to determine the chemical characterization and biological effects of these two *Anthemis* species. Phytochemical profiles of ethyl acetate (EA), methanol (MeOH) and aqueous extracts were performed by ultra-high-performance liquid chromatography coupled with electrospray ionization high resolution mass spectrometry (UHPLC-ESI/HRMS). The samples were assayed for evaluating antioxidant and enzyme inhibitory potential, as well.

Finally, considering both the traditional antianxiety effect of *A. tinctoria,* the relationships between anxiety and brain oxidative/inflammatory stress [19], alongside with the well-established multi-target protective effects exerted by flavonoid fraction, in the brain [20], we studied the putative protective role of *Anthemis* extracts in isolated rat cortex challenged with a neurotoxicity stimulus (K^+^ 60 mM). The influence of extract supplementation on the levels of specific biomarkers of oxidative stress and neurotransmission, including lactate dehydrogenase (LDH) and serotonin (5-HT), was investigated using validated analytical methods. An untargeted proteomic profile was also performed on rat cortex homogenate, in order to explore the putative mechanism of action of *Anthemis* extracts. It is expected that results presented in this study will support the protective effects of the studied *Anthemis* extracts as potential pharmacological agents.

## 2. Results and Discussion

### 2.1. Total Phenolic and Flavonoid Contents

Phenolic compounds are of increasing interest mainly due to their diverse chemical structure and wide biological activity valuable in the prevention of some chronic or degenerative diseases. To this end, the evaluation of the phytochemical profile of plant extracts is important. In the present study, the total phenol and flavonoid contents of EA, MeOH, and aqueous extracts of ATP and ACT were illustrated in Table 1. The phenolic content of ATP and ACT ranged from 26.46 to 100.09 mg GAE/g and 21.31 to 47.61 mg GAE/g, respectively. Highest phenolic content was observed in the MeOH extract of ATP, followed by its aqueous extract. Whilst for ACT, MeOH extract contained the highest amount of phenols, followed by EA extract. Regarding the total flavonoid content, the results showed that EA (ATP: 45.82 an ACT: 46.26 mg RE/g) and MeOH (ATP: 48.54 and ACT: 45.08 mg RE/g) extracts of both species were rich in flavonoids.

### 2.2. LC-MS Results

In the present study, 70 compounds were tentatively identified by UHPLC-ESI/MS in both ACT and ATP extracts. The negative ion mode was used for analysis of acylquinic acids and flavonoids, while positive ion mode was used for sesquiterpenes determination (Table 2).

#### 2.2.1. Acylquinic Acids

Twenty nine acylquinic acids were identified in tested *Anthemis* extracts (Table 2). The acylquinic acids elucidation was based on the hierarchical key developed by Clifford and colleagues [21,22]. Peaks **4**, **5**, **6**, and **7** were identified as 3-*O*-, 1-*O*-, 5-*O*- and 4-*O*-caffeoylquinic acids ([M − H]^−^ at *m/z* 353.088), respectively, according to the relative abundance of the characteristic fragment ions at *m/z* 191.055 [quinic acid − H]^−^, 179.034 [caffeic acid − H]^−^, 173.045 [quinic acid − H − H_2_O]^−^, and 135.044 [caffeic acid – H − CO_2_]^−^ [21,22]. Compounds **4** and **6** were identified by comparison with neochlorogenic and chlorogenic acid, respectively. In the same manner, peaks **1**, **2**, and **3** were assigned as 3-*O*-, 5-*O*-, and 1-*O*-*p*-coumaroylquinic acids ([M − H]^−^ at *m/z* 337.093), while peaks **8**, **9**, **10**, and **11** ([M − H]^−^ at *m/z* 367.103) were assigned as 3-*O*-, 1-*O*- 5-*O*-, and 4-*O*-feruloylquinic acid (Table 4). With respect to the diacylquinic acids, peaks **12**–**15** were related to 3,4-*O*-, 1,5-O, 3,5-*O*, and 4,5-*O*-dicaffeoylquinic acids ([M − H]^−^ at *m/z* 515.120); **12** and **13** were identified by comparison with standards. The presence of **14** was evidenced by the relative abundance of the ions at *m/z* 191.055, 179.034, and 135.043 [21,22,23], while the ion at 173.044 was prominent for **15**. Compounds **16**–**24**, [M − H]^−^ at *m/z* 529.136 were tentatively identified as caffeoylferuloylquinic acids [21,22]. Among the tricaffeoylquinic acids, peaks **25**–**29** were related to ([M − H]^−^ at *m/z* 677.152). Compounds **26**, **28**, and **29** yielded indicative fragment ions at 173.045 deduced 4-substituted CQA [22]. According to the presence of weak signal at *m/z* 203.034, the relative intensity of the fragment ion at *m/z* 335.078, and lipophilicity, peaks **26**, **28**, and **29** were tentatively assigned as 1,3,4-*O*-, 1,4,5-*O*-, and 3,4,5-*O*-tricaffeoylquinic acid, while **25** and **27** were related to 3,4,5-*O*-tricaffeoylquinic acid and its isomer.

#### 2.2.2. Flavonoids

Based on literature and comparison with standards, 15 flavonoid aglycones **30**–**44** (most of them methoxylated), twelve glycosides, and one caffeoyl-*O*-flavonoid were identified in the studied extracts (Table 4). Regarding **41**–**43** ([M − H]^−^ at *m/z* 345.061), the fragment ion at *m/z* 287.020, due to consecutive loss of 2CH_3_^−^ and CO is more intense in the product-ion spectra of **43** than **41** and **42**. Probably methoxylation of **43** in both A- and C-rings provides very stable fragments due to concurrent methyl loss [24]. Fragment ion at *m/z* 121.028 (^1,2^B) (for **41** and **42**) were attributed to the Retro-Diels Alder (RDA) cleavages of the flavonoid skeleton specific for 3′,4′-dihydroxy flavonols [25]. Thus, according to literature, **41**–**43** were tentatively identified as eupatolitin, spinatoside, and spinacetin, respectively.

The fragmentation fingerprints of **52** and **56** were associated with isorhamnetin derivatives, witnessed by the abundant fragment ion at *m/z* 315.051 supported by the ions at *m/z* 300.027 and 133.028 [24]. Fragmentation patterns and monoisotopic profiles of **52** was in good agreement with those of caffeoyl-*O*-isorhamnetin. The fragmentation of [M − H]^−^ at *m/z* 609.1472 (**56**) yielded abundant ion at *m/z* 315.0517 ([M − H − 294.095]^−^ indicating the loss of hexose and pentose moieties.

#### 2.2.3. Sesquiterpenes

Thirteen sesquiterpene lactones including one eudesmanolide, three germacranolides, and nine guaianolides, were tentatively identified in both ACT and ATP extracts. Concerning compound **58** ([M + H]^+^ at *m/z* 229.122), its fragmentation pattern involved losses of 18 Da (H_2_O), 28 Da (CO) and 46 Da (CO_2_H) suggesting chamazulene carboxylic acid, a degradation product of proazulenic sesquiterpene lactones, e.g., matricarin [25]. Similar fragmentation patterns were observed in spectra of **59** and **60.** In addition, a loss of 44 Da (CO_2_) and fragment ions at *m/z* 185.095 ([M + H – 16 − 44]^+^ and 95.049 (C_6_H_7_O) due to the overall fracture of lactone ring, suggested dehydroleucodin or isodehydroleucodin [26]. Accordingly, **61** was assigned as leucodin ([M + H]^+^ at *m/z* 247.132), where C-13 was saturated in a methyl group. **68** and **69** were tentatively identified as matricarin and its isomer, due to the concomitant loss of (CO_2_ + H_2_O) at *m/z* 245.117 from the additional acetyl group [3]. Three isobaric sesquiterpene lactones **63**–**65** shared the same [M + H]^+^ at *m/z* 263.127 (exact mass). Peaks **63**–**65** demonstrated difference of 15.995 Da, in comparison to **61**, suggesting the presence of an additional hydroxyl group. Thus **63**–**65** were tentatively assigned to hydroxyleucodin and its isomers [27]. In the same manner, peaks **62**, isobaric pair **66**/**67**, and **70** were ascribed as parthenolide, stizolin, and ludalbine, respectively, previously identified in *Anthemis* species [3].

### 2.3. Antioxidant Activity

Oxidative stress-related diseases often arise as a result of the imbalance between the production of free radicals and reactive oxygen/nitrogen species, and antioxidant defences. These diseases can be managed/prevented using natural antioxidants that represent promising therapeutic candidates [29]. Different antioxidant assays are needed to obtain certain information regarding antioxidant profile of herbal extracts. From this point, the antioxidant capacity of different extracts of ATP and ACT were evaluated using multiple assays based on different mechanisms and the results were presented in Table 3.

Based on the experimental results (Table 3), it can be noticed that for both species, MeOH extract exhibited the highest total antioxidant activity. According to data presented in Table 3, ATP MeOH extract (DPPH: 407.07 ± 8.88 and ABTS: 320.11 ± 5.67 mg TE/g), followed by the aqueous extract (DPPH: 298.40 ± 6.74 and ABTS: 303.16 ± 8.57 mg TE/g) showed higher radical scavenging activity in both assays. Likewise, ACT MeOH (DPPH: 97.22 ± 0.22 and ABTS: 112.41 ± 2.35 mg TE/g) and aqueous extracts (DPPH: 86.74 ± 2.46 and ABTS: 127.68 ± 0.45 mg TE/g) showed potent radical scavenging activity.

CUPRAC and FRAP assays were employed to assess the reducing capacity of different extracts. CUPRAC method evaluates the conversion of Cu (II) into Cu (I) while FRAP assay measures the reducing potential of an antioxidant reacting with the colourless TPTZ/Fe^3+^ complex to form a blue TPTZ/Fe^2+^ complex at low pH [30]. Remarkable reducing potencies were displayed by MeOH (CUPRAC: 691.17 ± 12.07 and FRAP: 362.12 ± 2.63) and aqueous (CUPRAC: 584.01 ± 8.71 and FRAP: 316.34 ± 4.15 mg TE/g) extracts of APT. EA extract displayed the lowest reducing capacity. This trend was also observed as regards ACT extracts (Table 3).

Chelation of pro-oxidant metals is recognized as one of the most important mechanisms of action of antioxidants. Particularly, iron is the most powerful and abundant pro-oxidant and transition metal which causes oxidative changes of cellular components, such as, lipids and proteins [31]. Evaluation of iron chelating activity showed that ATP and ACT extracts possessed notable chelation potential, with the highest activity displayed by ATP EA extract (39.01 ± 4.42 mg EDTAE/g) and ACT aqueous extract (39.64 ± 1.34 mg EDTAE/g).

### 2.4. Enzyme Inhibitory Activity

The enzyme inhibitory effect of ATP and ACT extracts was determined against cholinesterases, α-amylase, α-glucosidase, and tyrosinase and the results were presented in Table 4. EA and MeOH extracts of ATP and ACT exhibited potent inhibitory activity against AChE, with the highest activity observed for ATP MeOH extract (3.28 ± 0.43 mg GALAE/g) and ACT EA extract (4.68 ± 0.21 mg GALAE/g). On the other hand, ATP EA extract (3.48 ± 0.21 mg GALAE/g), ACT EA extract (2.51 ± 0.34 mg GALAE/g), and ACT MeOH extract (1.15 ± 0.05 mg GALAE/g) showed inhibitory effect against BChE. Both AChE and BChE hydrolyze acetylcholine and terminate the synaptic transmission [31]. While enhanced AChE activity is associated with early stages of AD, BChE activity is found to increase with the progression of the disease. Therefore, both enzymes are considered as legitimate therapeutic targets for managing AD [32,33].

Based on the results (Table 4), it can be observed that the extracts of both species showed remarkable inhibitory activity against tyrosinase with values ranging from 124.60 ± 0.15 to 72.10 ± 1.64 mg KAE/g and from 128.85 ± 1.41 to 88.95 ± 0.49 mg KAE/g for ATP and ACT, respectively. The lowest inhibitory activity against tyrosinase was detected for the aqueous extracts of both species. Tyrosinase is a key enzyme in melanin biosynthesis which is responsible for skin pigmentation. However, excessive melanin production could lead to various skin disorders such as melasma, lentigines, age spots, and post-inflammatory hyperpigmentation. Thus, tyrosinase inhibitors, used as hypopigmenting agents, became increasingly important for medicinal and cosmetic products [34].

Type II diabetes is a growing pandemic and poses an enormous public health challenge for almost every country worldwide. α-Amylase and α-glucosidase are considered as key therapeutic targets for the management of type II diabetes. α-Amylase and α-glucosidase are carbohydrate hydrolysing enzymes, responsible for the breakdown of carbohydrates into glucose [35,36]. The present study showed that all ATP and ACT extracts displayed weak α-amylase inhibitory effects, despite being active α-glucosidase inhibitors. The highest inhibitory effect against α-amylase (0.78 and 0.65 ACAEs/g extract, for ATP and ACT, respectively) and α-glucosidase (21.94 and 24.16 ACAEs/g extract, for ATP and ACT, respectively) was displayed by EA extracts.

### 2.5. Multivariate Analysis

**Figure 1:**
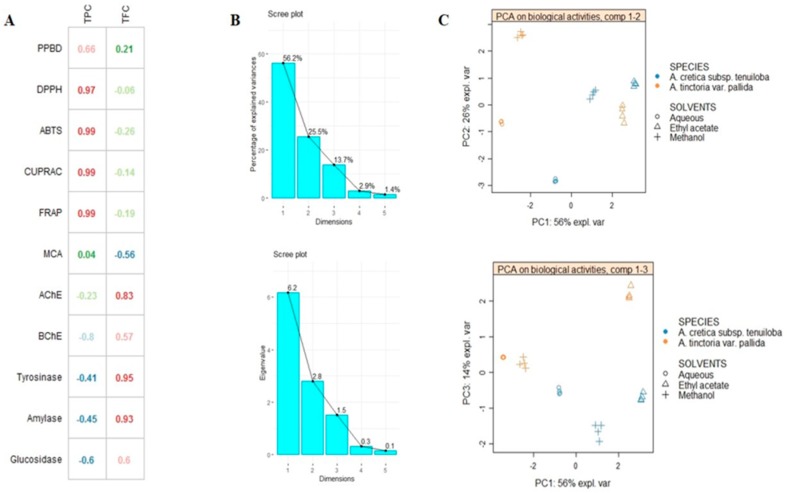
(**A**): Relationship between total phenol content (TPC), total flavonoid content (TFC) and biological activities. (**B**,**C**): results of preliminary multivariate analysis with PCA (**B**: Percentage of explained variance and Eigen value per component, **C**: PCA sample plot on PC1 vs. PC2 and PC1 vs. PC3 respectively).

In an attempt to compare the difference between extract activity, multivariate statistic (sPLS-DA) was carried out by using principal component analysis (PCA) and partial least squares projection to latent structures. PCA is very useful in reducing the dimension of data with minimum loss of information. Figure 1 showed the score plots of projected extracts on principal components PC1 vs. PC2 and PC1 vs. PC3. PCA allowed analysis of biological activities disparity between the extracts. The first three components defining most of variance were found to be the best to clearly classify the extracts (Figure 1C). Nevertheless, the result obtained did not facilitate us for a better discrimination and classification of extracts. Thus, it was considered necessary to apply sPLS-DA, a supervised multivariate analysis known to offer many advantages over PCA, notably by erecting more parsimonious and easily interpretable models compared to PCA.

**Figure 2:**
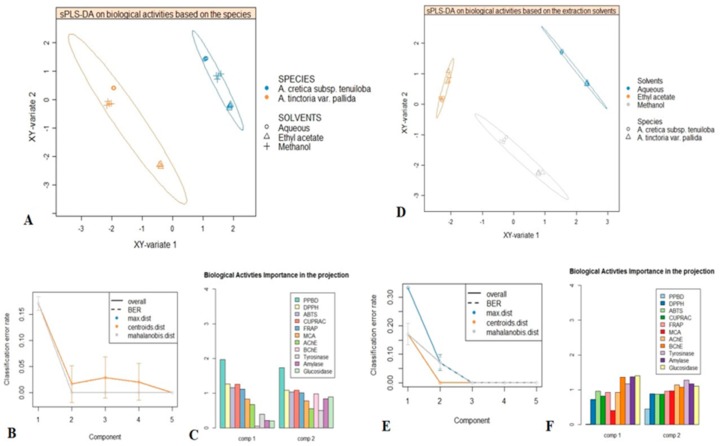
Supervised analysis with sPLS-DA. **A**: sPLS-DA samples plot with confidence ellipse plots considering the species as class membership criteria. **B**: Performance of the model (BER) for three prediction distances using 10 × 5-fold cross-validation. **C**: VIP score plot displaying the biological activities having highly contributed to the discrimination of both studied species. **D**: Factorial plan 1-2 of the sPLS-DA with confidence ellipse plots according to the extraction conditions as class membership criteria. **E**: The model performance per component for the three prediction distances using 5-fold cross-validation repeated 10 times. **F**: VIP score plot showing the biological activities outlining the difference between the three extraction conditions.

Firstly, analysed species were used as class membership criteria to assess whether they were characterized by distinctive biological activities. sPLS-DA samples plot was reported in Figure 2; as shown, a clear separation between *A. cretica* subsp. *tenuiloba* and *A. tinctoria* var. *pallida* was achieved, thus suggesting distinctive biological activities. Afterwards, with the aim to identify the most discriminant biological activities providing the differences overviewed in the sPLS-DA samples plot, VIP (variable importance in projection) plot was generated (Figure 2). Five biological activities including PPBD, DPPH, ABTS, CUPRAC and FRAP possessed a VIP score upper 1, which suggested them as discriminants for the two species.

Secondly, sPLS-DA was performed considering three different extraction conditions, in order to evaluate the effect of extraction solvents on biological activities. As shown in samples plot (Figure 2) the subspace formed by the first two components showed that methanol, water and ethyl acetate extracts were well separated. Next, the prediction performance and the number of components necessary for the final model were evaluated according to BER (Balanced Error Rate). The performance of our model reached its best for two components, which suggested ncomp = 2 for a final sPLS-DA model (Figure 2). Subsequently, the biological activities having highly contributed to the separation of used solvents were identified. As it could be seen in Figure 2, AChE, BChE, tyrosinase, α-amylase and α-glucosidase were the most contributing biological activities (Figure 2).

### 2.6. Multidirectional Biological Evaluation

**Figure 3:**
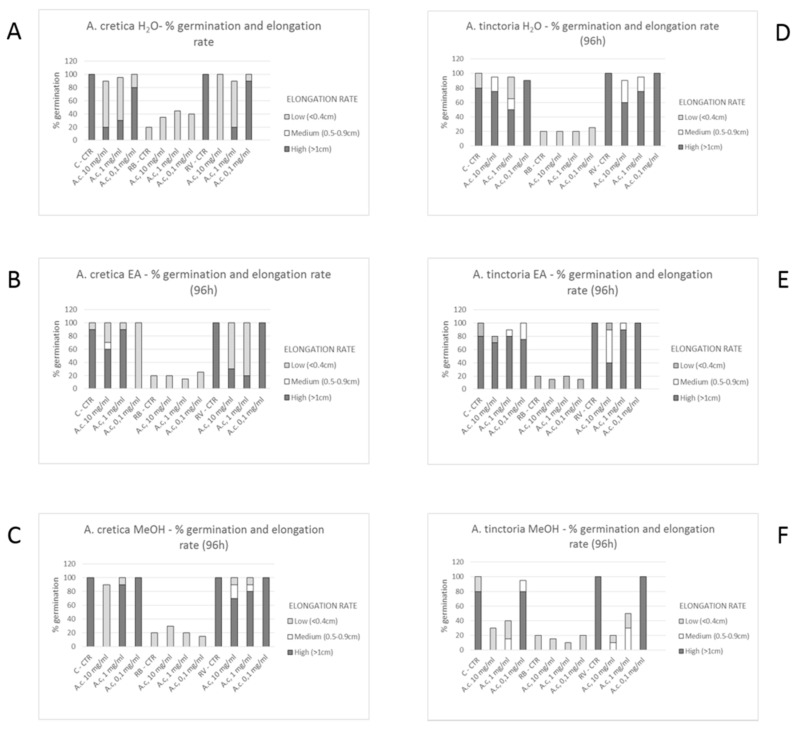
Seedling germination and growth of Canasta (C), Romana verde (RV) and Romana bionda (RB) seeds challenged with *A. tinctoria and A. Cretica* extracts. Results are expressed as root and hypocotyl (seedling) length ± SD at different concentrations and mean of GP after the fourth day since the sowing. (**A**): Effect of *A. cretica* water extract on seedling germination. (**B**): Effect of *A. cretica* ethyl acetate (EA) extract on seedling germination. (**C**): Effect of *A. cretica* water methanol (MeOH) on seedling germination. (**D**): Effect of *A. tinctoria* water extract on seedling germination. (**E**): Effect of *A. tinctoria* ethyl acetate (EA) extract on seedling germination. (**F**): Effect of *A. tinctoria* water methanol (MeOH) on seedling germination.

The biological activity of *Anthemis* extracts was formerly evaluated through allelopathy assay, a validated pharmacognostic test for discriminating herbal extract phytotoxicity. Particularly, we investigated the effects of scalar extract concentrations (100 µg/mL–10 mg/mL) on seedling germination of three commercial lettuce varieties, namely Canasta (C), Romana verde (RV), and Romana bionda (RB). After challenging the seeds with *Anthemis* water and EA extracts, we observed that germination process was unaffected in the tested concentration range (Figure 3A–E). Conversely, ATP MeOH extract displayed concentration-dependent inhibition of seedling germination, in the range 1–10 mg/mL (Figure 3F). The root elongation rate test revealed evident inhibitory effect, in the range 1–10 mg/mL. On the other hand, extracts resulted biocompatible at the lowest tested concentration (100 µg/mL), with percentage elongation rate ≥70% compared to vehicle untreated group. The results of elongation rate test suggest a further toxicological investigation, with independent methods in order to confirm the biocompatibility limit, as described below.

The potential toxicity of water, MeOH and EA extracts of *Anthemis* species (0.1–20 mg/mL) was also investigated through brine shrimp (*Artemia salina* Leach) lethality assay. Evaluation of lethality induced on brine shrimp, *Artemia salina* Leach, is considered predictive of cytotoxicity [37]. The results of this test revealed LC_50_ values < 10 mg/mL, for all tested extracts.

**Figure 4:**
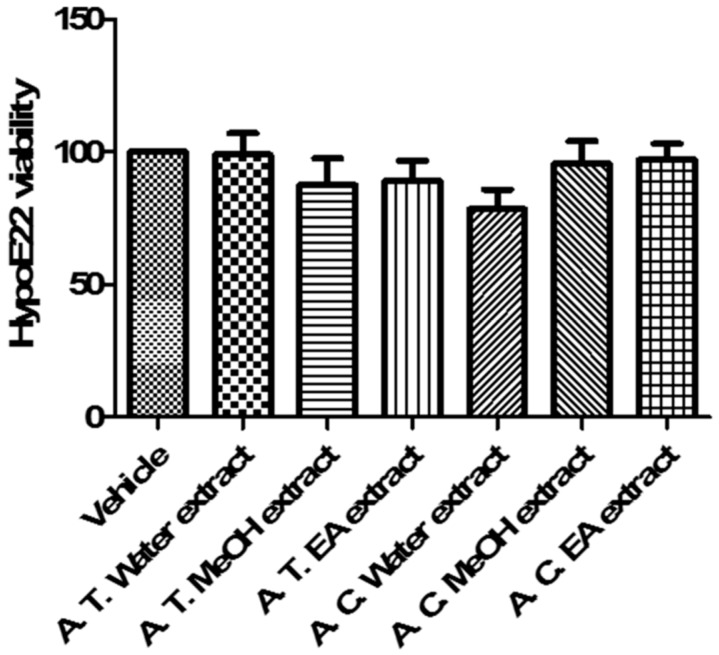
Effect of *A. tinctoria* (A. T.) and *A. cretica* (A. C.) extracts (100 µg/mL) on HypoE22 cell line viability (MTT test). Data are means ± SD of three experiments performed in triplicate.

Additionally, we evaluated the activity of *Anthemis* extracts on HypoE22 cell line viability. According to brine shrimp and allelopathy assays, we tested the extracts at 100 µg/mL. MTT test revealed that *Anthemis* extracts were well tolerated by HypoE22 cells, with a resulting cell viability ≥70% (Figure 4). This concentration was used for subsequent ex vivo investigations aimed to elucidate extract neuroprotective effects, as following reported.

**Figure 5:**
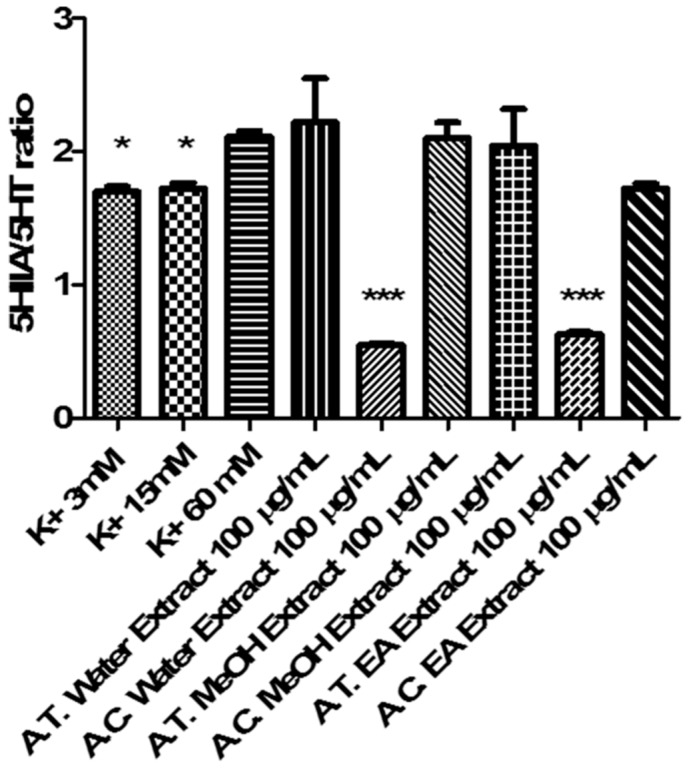
Effect of *A. tinctoria* (A. T.) and *A. cretica* (A. C.) extracts (100 µg/mL) on serotonin (5-HT) turnover, expressed as 5HIIA/5-HT ratio. Turnover was evaluated on isolated rat cortex challenged with basal (K^+^ 3mM) and depolarizing stimuli (K^+^ 15 mM; K^+^ 60 mM). Data are means ± SD of three experiments performed in triplicate. ANOVA, *p* < 0.0001; post-hoc, * *p* < 0.05, *** *p* < 0.001 vs. K^+^ 60 mM control group.

Cortical spreading depression (CSD) is a pathophysiological and mass depolarization of neurons and glial cells which is characterized by a change in ion and water distribution across neuron membrane associated with cytotoxic effects, including neuron death [38]. In physiological conditions, neurotransmitter release is elicited by depolarizing-stimuli (K^+^ 9–15 mM) which, through the increased passage of Ca^2+^ ions across nerve terminals via voltage-sensitive calcium channels (VSCCs), stimulates classical neurotransmitter exocytosis. On the other hand, in CSD, the neurotransmitter release increases possibly through additional mechanisms, including membrane transporter reversal [39]. Particularly, excitotoxicity depolarizing-stimuli (K^+^ ≥ 50 mM) were reported to increase significantly 5-HT overflow [40] which could stimulate neurotransmitter turnover, thus explaining the cortical 5-HT depletion induced by CSD, in vivo [41]. CSD has been recently described as a potential triggering mechanism in migraine with *aura*, via the activation of trigeminal nociceptive system, both peripherally and centrally [42]. While low 5-HT state could play a pivotal role in migraine attack, through multiple effects, including the reduction of pain perception threshold, the increased tendency of having headache and the interference with the control of cerebrovascular nociception [41]. A chronic reduction of 5-HT is also seen in migraineurs and in depressed patients, while amitriptyline and venlafaxine are first-choice drugs for treating patients suffering from migraine with comorbid depression [43]. Considering the role played by 5-HT in anxiety and migraine [41,43], and the traditional use of *Anthemis* species in anxiety [18], we tested ATP and ACT extracts (100 µg/mL) in isolated cortex specimens challenged with an excitotoxicity stimulus constituted by K^+^ (60 mM) Krebs-Ringer buffer. The results indicated that ATP and ACT EA extracts and ACT water extracts were able to completely blunt K+ (60 mM)-induced 5HIIA/5-HT ratio (Figure 5), which has long been considered as a valuable index of 5-HT degradation, in the brain [44,45]. On the other hand, MeOH extracts were ineffective in modifying K^+^ (60 mM)-induced 5-HT degradation (Figure 5). Conversely, MeOH extracts revealed a more selective enzyme inhibition on AChE (Table 4). Recently, pilocarpine, a muscarinic receptor agonist, was able to antagonize CSD effects, after sub-convulsing dose administration [46], thus suggesting a role played by acetylcholine signaling stimulation, in CSD. Actually, extract capacity to improve 5-HT and acetylcholine pathways could be related to their antiradical activity (Table 3) [47]. Previously, antioxidant herbal extracts were shown to blunt oxidative stress-induced reduction of neurotransmitter level, in the brain [48,49]. Specifically, water *Harpagophytum procumbens* extract was able to prevent cortex 5-HT depletion induced by amyloid β-peptide [48], possibly through concomitant antioxidant mechanisms, that have been, at least partially, displayed by *Anthemis* extracts, as well. Whereas multiple studies also pointed out the efficacy of isolated secondary metabolites, including polyphenols and tocopherols, in blunting oxidative stress-induced monoamine depletion, thus further suggesting a putative role in managing clinical symptoms related to neurodegenerative diseases [50,51].

**Figure 6:**
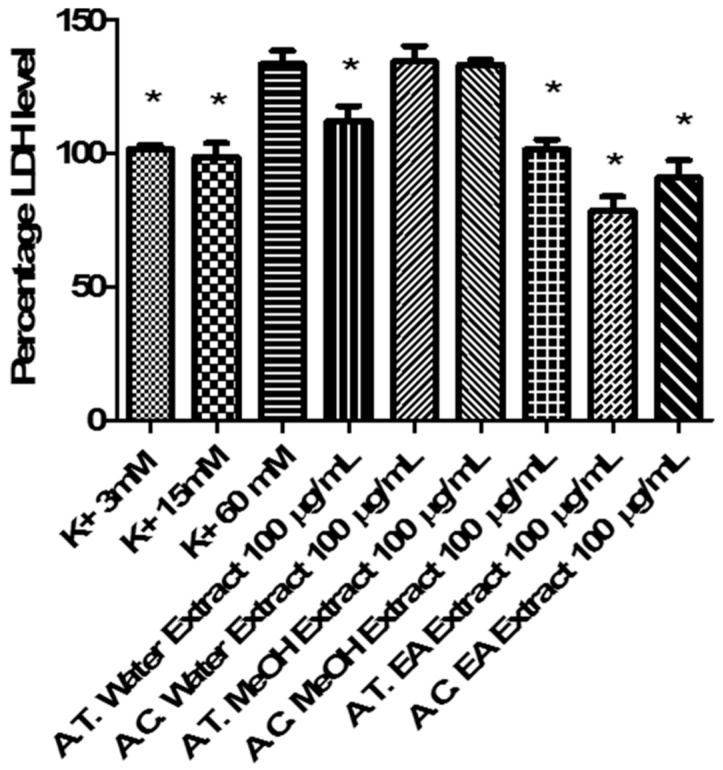
Effect of *A. tinctoria* (A. T.) and *A. cretica* (A. C.) extracts (100 µg/mL) on lactate dehydrogenase (LDH) level, measured on isolated rat cortex challenged with basal (K^+^ 3mM) and depolarizing stimuli (K^+^ 15 mM; K^+^ 60 mM). Data are means ± SD of three experiments performed in triplicate. ANOVA, *p* < 0.0001; post-hoc, * *p* < 0.05 vs. K^+^ 60 mM control group.

On the other hand, after evaluating the effects of *Anthemis* extracts (100 µg/mL) on LDH, a well-recognized marker of tissue damage [52], we observed that ACT EA and MeOH extracts, alongside with ATP water and EA extracts, revealed effective in blunting K^+^ (60 mM)-induced LDH level (Figure 6). Considering the results of qualitative fingerprint analysis, we could hypothesize that the observed effects might be related to the presence of flavonoids and terpenes such as apigenin, patuletin, jaceosidin, quercetin, luteolin, and parthenolide.

In agreement with the antiradical activity (Table 2) and blunting effect on 5-HT turnover (Figure 5), ACT water extract samples has been subjected to a further proteomic study, in order to deepen our knowledge about the putative mechanism of action related to neuroprotective effects. The deepening about ACT water extract was performed in comparison with the corresponding ATP extract that, despite showing a null effect on 5-HT turnover (Figure 5), displayed a significant inhibitory effect on K^+^ (60 mM)-induced LDH level (Figure 6).

**Figure 7:**
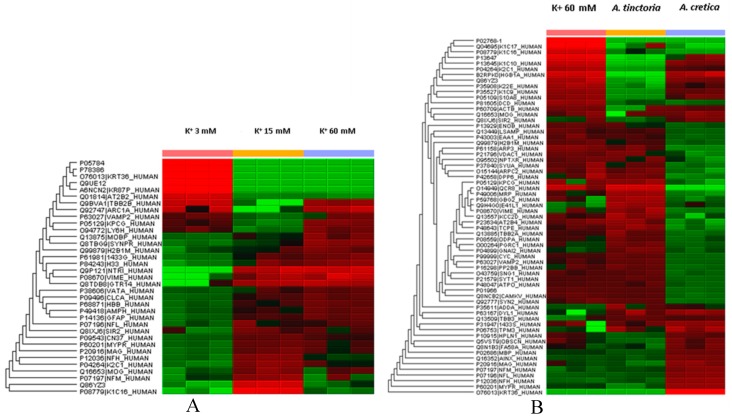
Panel **A**: Untargeted proteomic analysis performed on rat cortex challenged with basal (K+ 3mM) and depolarizing stimuli (K^+^ 15 mM; K^+^ 60 mM). The activity of the detected proteins was calculated in comparison with the calibrator of the experiment (K^+^ 60 mM). Panel **B**: Untargeted proteomic analysis showing the effects of *A. tinctoria* and *A. cretica* water extracts (100 µg/mL) on rat cortex challenged with excitotoxicity depolarizing stimulus (K^+^ 60 mM). T The activity of the detected proteins was calculated in comparison with the calibrator of the experiment (K^+^ 60 mM). In subfigure **A**, it is showed that K^+^ 60 mM depolarizing stimulus downregulated NEFMs and upregulated VAMP-2 and PKCγ levels. On the other hand, as depicted in subfigure **B**, *A. cretica* water extract (100 µg/mL) was able to restore the activity of specific proteins involved in neuron morphology and neurotransmission, including NEFMs, VAMP-2, and PKCγ. After treating rat cortex with that *A. cretica* water extract, the activity of these proteins was similar to that measured after challenging the brain tissue with physiologic depolarizing stimulus (K^+^ 15 mM).

Particularly, untargeted proteomic analysis showed that K^+^ 60 mM was able to significantly downregulate neurofilament (NFEM) proteins (Figure 7A/Supplementary Data 1), expressed along the axons and involved in axonal diameter regulation. Reduced NFEM levels have long been related to neurodegeneration [53]. The treatment of isolated rat cortex with ACT water extract was able to prevent NFEM downregulation, restoring the activity of NEFM proteins during K^+^ 15 mM physiologic depolarizing stimulus. While ATP water extract did not exert any relevant effect on NFEM level, in isolated rat cortex challenged with K^+^ 60 mM (Figure 7B/Supplementary Data 2). Conversely, K^+^ 60 mM stimulus led to significant upregulation of protein C kinase γ (PKCγ) and vesicle-associated membrane protein-2 (VAMP-2) (Figure 7A/Supplementary Data 2), compared to physiologic depolarizing stimulus (K^+^ 15 mM). VAMP-2 is placed on the membranes of neuronal endings’synaptic vesicles, playing a key role in synaptic vesicle fusion to the presynaptic neuronal ending membrane [54]. Multiple studies suggested upregulation of VAMP-2 level during hypoxia [55,56], which is strictly related to high K^+^ concentration-induced CNS injury [39]. PKCγ plays multiple roles in neuronal cells and eye tissues, such as regulation of the neuronal receptors GRIA4/GLUR4 and GRIN1/NMDAR1, modulation of receptors and neuronal functions related to sensitivity to opiates, pain and alcohol, mediation of synaptic function and cell survival after ischemia, and inhibition of gap junction activity after oxidative stress. Its level is positively related to migraine pathogenesis [57]. Additionally, PKCγ gene expression was observed in histidine triad nucleotide-binding protein 1 (Hint1) KO mice, that also showed increased anxiety-related behavior, compared to wild type control mice [58]. Also in this case, treatment of isolated rat cortex with ACT water extract was able to restore the activity of both VAMP-2 and PKCγ during K^+^ 15 mM-depolarizing stimulus (Figure 7A/Supplementary Data 2), further supporting the neuroprotective effects of this extract against the burden of oxidative stress and inflammation occurring in CSD.

## 3. Materials and Methods

### 3.1. Plant Material and Preparation of Extracts

Sampling of the plant species (*Anthemis tinctoria* L. var. *pallida* DC. and *A. cretica* L. subsp. *tenuiloba* (DC.) Grierson) was done in Kastamonu (Hanonu) of Turkey in 2018. The collected plant identity was confirmed by the botanist Dr. Ismail Senkardes (Marmara University, Faculty of Pharmacy, Istambul, Turkey). Aerial parts were collected from wild full blooming plants and dried in ventilated oven (in the dark, temperature 40 °C) until constant weight. Afterwards, dried materials were powdered.

Methanol and ethyl acetate extracts were prepared through maceration method (5 g plant material in 100 mL solvents for 24 h). After that, extracts were filtered and then concentrated by using one rotary evaporator under *vacuo*. Regarding water extracts, infusion method was selected (5 g plant was kept in 100 mL boiling water in 20 min). The infusions were filtered and then lyophilized. All extracts were stored at +4 °C, avoiding light exposure.

### 3.2. Assays for Total Phenolic and Flavonoids

With reference to our earlier report [59], total bioactive components namely total phenols (TPC) and flavonoids (TFC) were measured by spectrophotometric assays. Gallic acid was the standard for phenols, while rutin was selected for flavonoids.

### 3.3. Antioxidant and Enzyme Inhibition Assays

Antioxidant properties of *Anthemis* extracts were determined by different in vitro assays namely FRAP, CUPRAC, DPPH, ABTS, chelating and phospomolybdenum assays. Regarding enzyme inhibitory properties, some enzymes including tyrosinase, cholinesterase, α-amylase and α-glucosidase were selected. All experimental procedures were given in our earlier report [59].

### 3.4. UHPLC-ESI/HRMS Analysis

Neochlorogenic acid (3-CQA) (4), chlorogenic acid (5-CQA) (6), apigenin (30), luteolin (31), quercetin (35), isoquercitrin (49), hyperoside (50), luteolin-7-*O*-rutinoside (54), and rutin (55) were obtained from Extrasynthese (Genay, France). 3,4-*O*-diCQA (12), 3,4-*O*-diCQA (13), diosmetin (33), rhamnetin (37), isorhamnetin (38), luteolin-7-*O*-glucuronide (47), isorhamnetin-7-*O*-glucoside (51), and isorhamnetin-3-*O*-rutinoside (57) were purchased from PhytoLab (Vestenbergsgreuth, Germany).

The UHPLC-ESI/HRMS analyses were carried out on a Q Exactive Plus heated electrospray ionization (HESI-II) – high resolution mass spectrometer (HRMS) (ThermoFisher Scientific, Inc., Bremen, Germany) equipped with an ultra-high-performance liquid chromatography (UHPLC) system Dionex Ultimate 3000RSLC (ThermoFisher Scientific, Inc.) [60].

### 3.5. Statistical Analysis for Antioxidant and Enzyme Inhibitory Assays

To interpret data gathered, R version 3.5.1 software (The R Foundation, St. Louis, MO, USA) with corrplot and mixOmics packages was used to perform univariate and multivariate statistical analyses. One way analysis of variance (ANOVA) and Tukey’s post hoc test were employed to compare bioactive compounds and biological activities between the samples. Also, relationships between bioactive compounds and biological activities were evaluated by the estimation of Pearson’s correlation. For multivariate analysis, biological activities of samples were firstly analyses by PCA to pinpoint similarities or differences between samples. Then sPLS-DA was applied by using the species and different extraction condition as class memberships respectively. This allowed better comparison between the two studied species and gauged the effect of the different extraction solvents on biological activities.

### 3.6. Pharmacological Assays

#### 3.6.1. Allelopathy Bioassay

Allelopathy bioassay was carried on the seeds of three commercial lettuces [Canasta (C), Romana verde (RV) and Romana bionda (RB)], because of their fast germination rate and high sensitivity. The detailed procedure has been extensively reported in our recent paper [61]. Seeds were treated with scalar *Anthemis* extract concentrations (0.1–10 mg/mL) and considered germinated for observed root length ≥ 1 mm, after the third day of treatment.

#### 3.6.2. *Artemia salina* Lethality Bioassay

*Artemia salina* lethality bioassay was performed as previously reported [61]. Brielfy, brine shrimp larvae were bred at 25–28 °C for 24h in presence of *Anthemis* extracts (0.1–20 mg/mL) dissolved in incubation medium (artificial sea water). After incubation period (24 h) with extracts, the number of surviving shrimps was evaluated and their vitality was compared to untreated control group. Experiments were carried out in triplicate, and percentage mortality was calculated with the following equation: ((T − S)/T) × 100, where T and S are the total number of incubated larvae and survival napulii, respectively.

#### 3.6.3. In Vitro Studies

Rat hypothalamic Hypo-E22 cells were cultured in DMEM (Euroclone), as previously reported [48]. The effects of *Anthemis* extracts (100 μg/mL) on Hypo-E22 cell line viability was evaluated through 3-(4,5-dimethylthiazol-2-yl)-2,5-diphenyltetrazolium bromide (MTT) test.

#### 3.6.4. Ex Vivo Cortical Spreading Depression Paradigm

Male adult Sprague-Dawley rats (200–250 g) were sacrificed by CO_2_ inhalation (100% CO_2_ at a flow rate of 20% of the chamber volume per min) and cortex specimens were immediately collected and maintained in thermostatic shaking bath at 37 °C for 1 h (incubation period), in Krebs-Ringer buffer at different K^+^ concentrations, as described below:

K^+^ 3 mM: corresponding to basal condition;
K^+^ 15 mM: corresponding to physiologic depolarizing-stimulus;
K^+^ 60 mM: corresponding to excitotoxicity depolarizing-stimulus.

The present experimental paradigm reproduced the neural pathophysiological condition named cortical spreading depression (CSD), and was designed according to previous ex vivo and in vivo studies, describing the use of elevated K^+^ concentrations (up to 50–60 mM) to induce central nervous system (CNS) injury [38,39,40]. During incubation, cortex specimens were challenged with water, MeOH and EA *A. tinctoria* and *A. cretica* extracts (100 μg/mL). Afterwards, individual cortex slices were homogenized in perchloric acid solution (0.05 M) in order to extract and quantify serotonin (5-HT) and its main metabolite (5-hydroxyindoleacetic acid, 5HIIA) via HPLC coupled to electrochemical detection, as previously reported [61,62]. The results were expressed as ng/mg wet tissue. Additionally, we carried out colorimetric evaluation of LDH level [52]. Finally, an untargeted proteomic profile was performed on rat cortex homogenate, as described below, in order to further elucidate the putative mechanism of action of *Anthemis* extracts.

### 3.7. Protein Extraction and Filter-aided Sample Preparation

After protein quantification, a volume corresponding to 50 ug of proteins was loaded onto a Nanosep 10-kDa-cutoff filter (Pall Corporation, Michigan city, MI, USA) and digested according to the protocol we routinely use in our laboratory. Briefly, the sample was washed twice with 200 µL urea buffer (8 M urea, 100 mM Tris pH 8.5 in milliQ water) to remove the detergents present in the lysis buffer. The proteins on the filter where subsequently reduced and alkylated by adding 100 µL of DTT solution (8 mM dithiothreitol in urea buffer) and 100 µL of IAA solution (50 mM iodoacetamide in Urea buffer). For protein digestion, the buffer was exchanged with 50 mM ammonium bicarbonate, before adding trypsin to a ratio of 1:50 (enzyme:substrate). The reaction was incubated for 16 h at 37 °C, and the mixture of peptides was collected by centrifugation, acidified with 10% trifluoroacetic acid and stored at −20 °C until analysis. The detailed description of mass spectrometric analysis is reported as “Supplementary Proteomic Analysis”.

### 3.8. Statistical Analysis for Pharmacological Assays

Statistical analysis was performed using GraphPad Prism version 5.01 for Windows (GraphPad Software, San Diego, CA, USA). Means ± S.E.M. were determined for each experimental group and analyzed by one-way analysis of variance (ANOVA), followed by Newman-Keuls comparison multiple test. Statistical significance was set at *p* < 0.05. As regards the animals randomized for each experimental group, the number was calculated on the basis of the “Resource Equation” N = (E + T)/T (10 ≤ E ≤ 20; https://www.nc3rs.org.uk/experimental-designstatistics).

## 4. Conclusions

**Figure 8:**
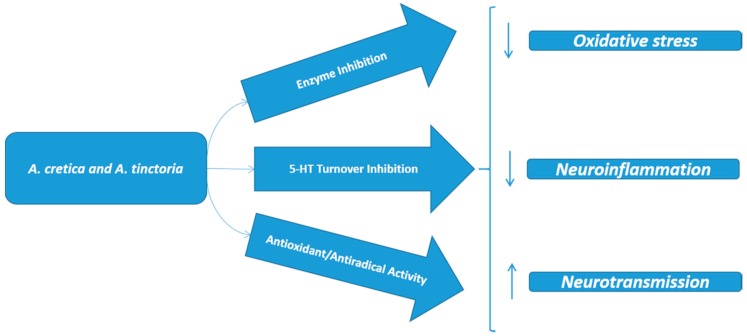
Protective effects induced by *A. cretica* and *A. tinctoria* extracts, as evidenced by the present pharmacological investigation.

Results collected in the present study indicated the promising biological effects of ATP and ACT extracts. As summarized in Figure 8, tested extracts showed significant antioxidant activity and potent inhibitory effects against key enzymes, involved in Alzheimer’s disease, type II diabetes, and hyperpigmentation conditions. Particularly, EA and methanol extracts of both species showed higher enzyme inhibitory activity (at least 1.5 fold: Table 4) compared to water extracts. Conversely, ACT water extract revealed more significant protective effects, as evidenced by reduced (−74%) cortex 5-HT turnover and restored activity of key proteins (i.e., NFEMs and PKCγ) involved in neuron morphology and neurotransmission, in the selected model of neurotoxicity. In this context *A. cretica* water extract appears to be a good candidate for future investigations aimed to confirm and characterize the observed pharmacological effects, possibly through the use of independent experimental methods.

## Figures and Tables

**Table 1:** Total phenol and flavonoid content of *Anthemis* extracts *.

Plant Names	Solvents	Total Phenol Content (mg GAE/g)	Total Flavonoid Content (mg RE/g)
*A. tinctoria* var. *pallida*	EA	26.46 ± 1.11 ^d^	45.82 ± 0.40 ^b^
	MeOH	100.09 ± 2.83 ^a^	48.54 ± 0.57 ^a^
	Aqueous	86.74 ± 1.80 ^b^	23.10 ± 0.13 ^d^
*A. cretica* subsp. *tenuiloba*	EA	21.31 ± 1.58 ^e^	46.26 ± 0.25 ^b^
	MeOH	46.73 ± 0.80 ^c^	45.08 ± 0.26 ^c^
	Aqueous	47.61 ± 1.89 ^c^	21.17 ± 0.24 ^e^

* Values expressed are means ± S.D. of three parallel measurements. GAE: Gallic acid equivalent; RE: Rutin equivalent. Different letters indicate significant differences in the extracts (*p* < 0.05).

**Table 2:** Peak assessment of compounds in *Anthemis* extracts.

Peak №	Accurate Mass [M − H]^−^ *m/z*	Molecular Formula	MS/MS Data *m/z*	t_R_ min	Exact Mass [M − H]^−^ *m/z*	Delta ppm	Tentative Structure	Ref.
**Acylquinnic acids**								
**Monoacylquinic acids**								
**1**	337.0946	C_16_H_17_O_8_	337.0946 (3.1), 191.0557 (7.7), 173.0453 (2.5), 163.0390 (100), 119.0488 (22.8)	3.05	337.0929	5.071	3-*p*-coumaroyl-quinic acid ^1,2,6^	[19]
**2**	337.0932	C_16_H_17_O_8_	337.0932 (7.2), 191.0554 (100), 173.0445 (6.7), 163.0390 (5.8), 119.0489 (5.5), 93.0329 (17.6)	4.07	337.0929	0.829	5-*p*-coumaroyl-quinic acid ^1,2,^^3,5,^^6^	[19]
**3**	337.0930	C_16_H_17_O_8_	337.0930 (5.9), 191.0554 (100), 173.0446 (1.9), 163.0391 (2.4), 127.0391 (1.5), 119.0487 (1.4), 111.0439 (1.3), 93.0331 (5.3), 85.0280 (7.1)	4.74	337.0929	0.473	1-*p*-coumaroyl-quinic acid ^1,2,^^5,^^6^	[19]
**4**	353.0879	C_16_H_17_O_9_	353.0879 (32.4), 191.0553 (100), 179.0341 (60.7), 173.0443 (4.1),161.0233 (3.9), 135.0439 (50.7), 111.0438 (0.9), 93.0333 (4.5), 85.0279 (8.7)	2.47	353.0867	0.325	neochlorogenic (3-caffeoylquinic) acid ^1,2,3,4,6^	*
**5**	353.0879	C_16_H_17_O_9_	353.0879 (29.9), 191.0554 (47.7), 179.0341 (70.1), 173.0447 (100), 135.0439 (57.7), 111.0436 (3.3), 93.331 (22.6), 85.0280 (8.6)	2.48	353.0867	0.240	1-caffeoylquinic acid ^1,2,3,4,6^	[19]
**6**	353.0880	C_16_H_17_O_9_	353.0880 (3.8), 191.0554 (100), 179.0338 (1.4), 173.0446 (0.9), 161.0234 (2.1), 135.0441 (1.1), 111.0435 (1.2), 93.0331 (2.7), 85.0279 (0.4)	3.30	353.0867	0.495	chlorogenic (5-caffeoylquinic) acid ^1,2,3,4,5,6^	*
**7**	353.0880	C_16_H_17_O_9_	353.0880 (28.1), 203.6057 (0.1), 191.0554 (100), 179.0341 (58.3), 173.0446 (4.6), 161.0233 (3.9), 135.0438 (51.8), 111.0437 (2.2), 93.0330 (4.82), 85.0279 (10.2)	5.92	353.0867	0.665	4-caffeoylquinic acid ^1,2,3,4,6^	[19]
**8**	367.1043	C_17_H_19_O_9_	367.1043 (12.5), 193.0500 (100), 173.0453 (3.9), 149.0598 (2.9), 134.0361 (65.7), 127.0395 (1.0), 111.0439 (1.4), 93.0331 (2.9)	3.54	367.1034	2.410	3-feruloylquinic acid ^1,2,^^5^^,6^	[19]
**9**	367.1039	C_17_H_19_O_9_	367.1039 (49.3), 161.0234 (100), 127.0390 (1.7), 85.0281 (13.4)	3.97	367.1034	1.238	1-feruloylquinic acid ^1,2,^^5^^,6^	[19]
**10**	367.1035	C_17_H_19_O_9_	367.1035 (14.1), 193.0499 (6.7), 191.0555 (100), 173.0447 (18.2), 134.0447 (10.9), 111.0437 (3.9), 93.0331 (26.2), 85.0280 (5.3)	4.52	367.1034	−0.015	5-feruloylquinic acid ^1,2,^^3,4,5^^,6^	
**11**	367.1030	C_17_H_19_O_9_	367.1030 (11.8), 193.0499 (17.1), 173.0446 (100), 111.0435 (3.1), 93.0331 (24.0)	4.78	367.1034	-1.322	4-feruloylquinic acid ^1,2,^^5^^,6^	[19]
**Diacylquinic acids**								
**12**	515.1196	C_25_H_23_O_12_	515.1196 (100), 353.0880 (18.5), 335.0788 (6.4), 203.0331 (1.3), 191.0554 (29.5), 179.0341 (58.1), 173.0446 (65.7), 161.0235 (19.8), 135.0439 (57.5), 127.0389 (3.6), 111.0436 (4.7), 93.0330 (18.7), 85.0277 (4.3)	5.79	515.1184	0.137	3,4-dicaffeoylquinic acid ^1,2,3,4,5,6^	*
**13**	515.1199	C_25_H_23_O_12_	515.1199 (21.8), 353.0881 (83.1), 335.0782 (2.6), 191.0554 (100), 179.0341 (48.6), 173.0445 (10.6), 161.0233 (12.4), 135.0439 (57.5), 127.0387 (2.8), 93.0332 (6.4), 85.0280 (9.0)	5.96	515.1184		1,5-dicaffeoylquinic acid ^1,2,3,4,5,6^	*
**14**	515.1204	C_25_H_23_O_12_	515.1204 (22.6), 353.0880 (87.4), 191.0555 (100), 179.0342 (52.0), 173.0455 (9.8), 161.0235 (8.9), 135.0438 (51.7), 93.0333 (2.8), 85.0281 (8.6),	6.14	515.1184	1.787	3,5-dicaffeoylquinic acid ^3,4^	[28]
**15**	515.1199	C_25_H_23_O_12_	515.1199 (75.6), 353.0880 (60.6), 203.0343 (3.1), 191.0555 (34.7), 179.0342 (72.3), 173.0447 (100), 135.0440 (48.1), 127.0384 (1.2), 111.0439 (3.7), 93.0330 (17.2), 85.0280 (4.2)	6.34	515.1184	0.720	4,5-dicaffeoylquinic acid ^1,2,3,4,5,6^	[28]
**16**	529.1356	C_26_H_25_O_12_	529.1356 (100), 367.1037 (8.3), 353.0889 (7.4), 349.0935 (5.2), 335.0774 (11.8), 193.0499 (60.5), 191.0555 (9.2), 179.0342 (42.1), 173.0446 (48.1), 161.0235 (24.6), 149.0596 (1.1), 134.0361 (56.0), 111.0437 (8.9), 93.0331 (14.7), 85.0276 (2.7)	6.00	529.1351	0.889	3-feruloyl-5-caffeoylquinic acid ^1,2,4,5,6^	[28]
**17**	529.1357	C_26_H_25_O_12_	529.1357 (55.4), 367.1038 (25.6), 193.0499 (2.6), 179.0342 (3.0), 173.0449 (1.9), 161.0234 (100), 135.0441 (12.7), 134.0367 (4.3), 127.0380 (0.5), 93.0331 (1.1), 85.0279 (3.2)	6.92	529.1351	1.003	1-feruloyl-5-caffeoylquinic acid ^1,2,3,5^	[28]
**18**	529.1354	C_26_H_25_O_12_	529.1354 (38.3), 367.1040 (39.6), 353.0883 (44.99), 193.0506 (15.6), 191.0555 (100), 179.0343 (41.61), 173.0454 (14.8), 161.0239 (13.0), 135.0440 (52.3), 134.0363 (21.5), 93.0332 (14.2), 85.0281 (7.4)	7.00	529.1351	0.436	3-caffeoyl-5-feruloylquinic acid ^1,2,3,4^	[28]
**19**	529.1352	C_26_H_25_O_12_	529.1352 (66.9), 367.1044 (100), 193.0504 (12.1), 179.0333 (57.2), 173.0447 (76.9), 161.0236 (15.6), 135.0439 (73.7), 134.0365 (49.6), 93.0331 (76.7)	7.12	529.1351	0.077	4-feruloyl-5-caffeoylquinic acid ^4,5,6^	[28]
**20**	529.1361	C_26_H_25_O_12_	529.1361 (14.2), 367.1036 (60.3), 193.0499 (14.5), 173.0447 (100), 161.0239 (1.1), 134.0362 (17.5), 127.0392 (1.0), 111.0436 (3.4), 93.0330 (24.9),	7.23	529.1351	1.816	3-caffeoyl-4-feruloylquinic acid ^1,2,3,6^	[28]
**21**	529.1388	C_26_H_25_O_12_	529.1388 (83.7), 353.0898 (56.5), 191.0550 (75.9), 179.0346 (79.9), 173.0448 (100), 161.0237 (11.9), 135.0439 (69.8), 93.0330 (38.2)	7.28	529.1351	6.880	4-caffeoyl-5-feruloylquinic acid ^4,5,6^	[28]
**22**	529.1356	C_26_H_25_O_12_	529.1356 (74.2), 367.1031 (21.5), 349.0925 (2.1), 191.0554 (1.2), 179.0341 (30.5), 173.0446 (4.94), 161.0234 (100), 135.0439 (49.6), 93.0331 (1.9)	7.34	529.1351	0.776	1-feruloyl-3-caffeoylquinic acid ^1,2,3,5^	[28]
**23**	529.1348	C_26_H_25_O_12_	529.1348 (100), 367.1052 (45.3), 179.0348 (10.3), 173.0449 (60.2), 161.0237 (24.0), 135.0441 (16.4), 111.0439 (13.0), 93.0331 (77.1)	7.44	529.1351	−0.717	4-feruloyl-?-caffeoylquinic acid ^4,6^	**
**24**	529.1353	C_26_H_25_O_12_	529.1353 (74.9), 367.1037(19.5), 349.0932 (4.4), 179.0341 (73.22), 161.0234 (62.3), 135.0439 (100), 134.0364 (12.4), 93.0331 (0.6), 85.0276 (0.5)	7.71	529.1351	0.304	1-feruloyl-3-caffeoylquinic acid-isomer ^1,2,5,6^	**
**Triacylquinic acids**								
**25**	677.1523	C_34_H_29_O_15_	677.1523 (2.9), 515.1105 (44.5), 353.0903 (3.7), 341.0888 (4.4), 191.0552 (9.6), 179.0341 (100), 173.0446 (6.0), 161.0230 (4.6), 135.0439 (79.8), 111.0443 (1.0), 93.0331 (1.6)	4.85	677.1512	1.634	1,3,5-tricaffeoylquinic acid ^1,2,4,5,6^	[28]
**26**	677.1524	C_34_H_29_O_15_	677.1524 (89.2), 515.1190 (17.3), 353.0879 (25.83), 341.0876 (8.0), 335.0772 (23.0), 323.0791 (5.1), 203.1523 (0.9), 191.0552 (30.6), 179.0341 (71.0), 173.0446 (54.7), 161.0233 (46.0), 135.0439 (100), 127.0388 (4.3), 111.0437 (7.8), 93.0330 (19.3), 85.0280 (1.9)	5.26	677.1512	1.812	1,3,4-tricaffeoylquinic acid ^1,2,3,5,6^	[28]
**27**	677.1529	C_34_H_29_O_15_	677.1529 (52.3), 515.1200 (55.7), 353.0878 (26.0), 341.0900 (15.6), 191.0553 (57.3), 179.0338 (75.3), 173.0449 (31.1), 161.0236 (34.66), 135.0439(100), 93.0328 (7.5)	5.37	677.1512	2.447	1,3,5-tricaffeoylquinic acid ^2,5,6^ isomer	**
**28**	677.1524	C_34_H_29_O_15_	677.1524 (87.1), 515.1281 (24.3), 353.0878 (26.2), 341.0880 (31.5), 335.0787 (2.3), 323.0779 (10.6), 191.0555 (34.4), 179.0342 (94.0), 173.0447 (78.7), 161.0235 (21.9), 135.0439 (100), 111.0439 (2.2), 93.0331 (24.8), 85.0279 (4.8)	5.66	677.1512	1.812	1,4,5-tricaffeoylquinic acid ^1,2,5,6^	[28]
**29**	677.1519	C_34_H_29_O_15_	677.1519 (88.4), 515.1203 (30.3), 353.0881 (55.6), 335.0779 (19.1), 203.0344 (1.3), 191.0554 (57.4), 179.0341 (79.9), 173.0447 (100), 161.0234 (33.1), 135.0439 (95.5), 111.0437 (1.1), 93.0331 (28.3), 85.0278 (6.3)	7.85	677.1512	0.985	3,4,5-tricaffeoylquinic acid ^1,2,3,4,5^	
**Flavonoids**								
**30**	269.0457	C_15_H_9_O_5_	269.0457 (100), 151.0027 (6.39), 149.0233 (5.74), 117.0332 (22.24), 107.0124 (5.35)	8.73	269.0444	0.644	apigenin ^1,2,3,4,5,6^	*
**31**	285.0406	C_15_H_9_O_6_	285.0406 (100), 151.0028 (5.92), 133.0282 (25.08), 107.0126 (3.32)	7.82	285.0393	0.452	luteolin ^1,2,3,4,5,6^	*
**32**	287.0566	C_15_H_11_O_6_	287.0566 (14.91), 151.0025 (100), 135.0435 (89.99), 125.0231 (5.03), 107.0124 (13.58)	7.53	287.0550	1.842	eriodictyol ^1,2,3,4,5,6^	[24]
**33**	299.0561	C_16_H_11_O_6_	299.0561 (58.00), 284.0328 (100), 256.0382 (0.86), 227.0350 (3.45), 211.0393 (2.22)	8.91	299.0550	0.029	diosmetin ^1,2,3,4,5,6^	*
**34**	299.0562	C_16_H_11_O_6_	299.0562 (95.99), 284.9335 (100), 227.047 (2.78), 151.0033 (3.67), 107.0123 (2.84)	9.09	299.0550	0.430	3,4′,7-trihydroxy-3′-methoxyflavone ^1,2,3,4,5,6^	[23]
**35**	301.0352	C_15_H_9_O_7_	301.0352 (100), 300.0273 (24.24), 178.9976 (21.40), 151.0025 (49.72), 121,0282 (16.11), 107.0124 (14.15)	7.83	301.0342	−0.717	quercetin ^1,2,3,4,5,6^	*
**36**	315.0512	C_16_H_11_O_7_	315.0512 (61.55), 300.0279 (100), 271.0252 (28.24), 255.030 (11.23), 227.0349 (2.28), 136.9872 (2.51),	8.34	315.0499	0.584	nepetin ^4,5,6^	Mass bank
**37**	315.0513	C_16_H_11_O_7_	315.0513 (86.15), 301.0315 (11.76), 300.0276 (100), 243. 0303 (0.70), 165.9890 (1.63), 136.9868 (9.78)	7.90	315.0499	0.965	rhamnetin ^1,2,3,4,5,6^	*
**38**	315.0514	C_16_H_11_O_7_	315.0514 (100), 301.0316 (3.73), 300.0273 (41.59), 243. 0298 (1.08), 151.0025 (7.85), 107.0126 (6.32)	9.26	315.0499	1.156	isorhamnetin ^1,2,3,4,5,6^	*
**39**	329.0670	C_17_H_13_O_7_	329.0607 (14.45), 314.0436 (100), 299.0198 (25.08), 271.0250 (47.23),133.0282 (5.34), 107.2971 (0.52)	9.25	329.0655	0.954	jaceosidin ^1,3,4,6^	Mass bank
**40**	331.0463	C_16_H_11_O_8_	331.0463 (100), 316.0226 (56.40), 287.0199 (15.97), 271.0246 (5.47), 270.0176 (4.09), 165.9897 (19.03)	7.80	331.0448	1.086	patuletin ^1,3,4,6^	[23]
**41**	345.0618	C_17_H_13_O_8_	345.0618 (91.18), 330.0384 (100), 315.0150 (50.33), 287.0201 (15.30), 121.0280 (1.86)	8.36	345.0604	0.694	eupatuletin ^4,5,6^	**
**42**	345.0618	C_17_H_13_O_8_	345.0618 (100), 330.0385 (95.66), 315.0150 (46.41), 287.0198 (14.78), 121.0284 (7.72)	8.40	345.0604	0.694	spinatoside ^1,2,3^	[23]
**43**	345.0619	C_17_H_13_O_8_	345.0619 (100), 330.0385 (42.35), 315.0145 (4.01), 301.0388 (6.46), 287.0199 (40.72)	9.38	345.0604	0.694	spinacetin ^1,2,3^	[23]
**44**	359.0775	C_18_H_15_O_8_	359.0775 (100), 344.0539 (49.89), 329.0304 (52.64), 301.0359 (6.67), 287.0139 (4.46)	9.95	359.0761	0.750	jaceidin ^1,2,3,4,5,6^	[23]
**45**	431.0981	C_21_H_19_O_10_	431.0981 (100), 269.0440 (27.72), 268.0378 (57.01)	6.17	431.0972	−0.673	apigenin-7-*O*-glucoside ^1,2,3,4,5,6^	*
**46**	447.0934	C_21_H_19_O_11_	447.0934 (100), 327.0507 (0.95), 285.0405 (99.96), 151.0030 (7.20), 133.0280 (5.74), 107.0123 (4.57)	5.45	447.0921	0.348	luteolin-7-*O*-glucoside ^1,2,3,4,5,6^	*
**47**	461.0725	C_21_H_17_O_12_	461.0725 (42.84), 285.0406 (100), 151.0025 (3.72), 133.0280 (10.72), 107.0121 (1.50)	5.46	461.0714	−0.150	luteolin-7-*O*-glucuronide ^1,2,3^	*
**48**	461.1094	C_22_H_21_O_11_	461.1094 (100), 446.0858 (28.76), 299.0554 (12.48), 284.0313 (9.73), 283.0250 (21.41), 269.0467 (2.08), 255.0300 (73.76), 227.0345 (0.67), 151,0028 (0.86)	6.37	461.1078	2.220	diosmetin-*O*-glucoside ^1,2,3^	**
**49**	463.0887	C_21_H_19_O_12_	463.0887 (100), 372.1873 (4.84), 301.0360 (93.71), 300.0282 (27.20)	4.77	463.0871	1.038	isoquercitrin ^1,2,3,4,5,6^	*
**50**	463.0903	C_21_H_19_O_12_	463.0903 (100), 301.0349 (47.50), 300.0276 (66.34)	5.29	463.0871	4.601	hyperoside ^1,2,3,4,5,6^	*
**51**	477.1041	C_22_H_21_O_12_	477.1041 (100), 315.0496 (14.04), 314.0434 (53.04), 300.0274 (5.68), 285.0411 (6.84), 243.0297 (22.73), 271.0249 (27.98), 151.0032 (3.66)	6.10	477.1027	0.442	isorhamnetin-7-*O*-glucoside ^1,2,3,4,5,6^	*
**52**	493.0777	C_25_H_17_O_11_	493.0777 (11.58), 315.0512 (99.41), 314.0435 (100), 300.0277 (14.30), 285.0411 (13.30), 243.0293 (34.06), 227.0341 (4.04), 177.0182 (16.10), 151.0030 (4.86), 133.0283 (12.50)	9.12	493.0765	0.194	caffeoyl-*O*-isorhamnetin ^1,3^	**
**53**	493.0974	C_22_H_21_O_13_	493.0974 (100), 331.0463 (96.71), 316.0224 (22.61), 287.0196 (26.93), 271.0253 (9.58), 165.9891 (8.24)	5.63	493.0976	−0.257	patuletin-*O*-hexoside ^4,5,6^	**
**54**	593.1504	C_27_H_29_O_15_	593.1504 (97.39), 318.6214 (6.24), 285.0405 (100), 284.0327 (60.74), 227.0352 (29.58)	5.71	593.1500	−1.354	luteolin-7-*O*-rutinoside ^1,2,3,4,5,6^	*
**55**	609.1467	C_27_H_29_O_16_	609.1467 (72.52), 343.0477 (1.65), 301.0354 (100), 300.0278 (38.67), 178.9970 (1.59), 151.0027 (13.46),121.0279 (1.84), 107.0123 (4.76)	5.17	609.1450	0.923	rutin ^1,2,3,4,5,6^	*
**56**	609.1472	C_27_H_29_O_16_	609.1472 (96.84), 411.8945 (5.23), 315.0517 (100), 300.0275 (48.87), 133.0284 (7.86)	5.43	609.1450	2.149	isorhamnetin-*O*-pentosyl-hexoside ^4,5,6^	**
**57**	623.1613	C_28_H_31_O_16_	623.1613 (5.57), 315.0514 (100), 301.0306 (1.22), 300.0279 (16.19), 151.0025 (7.85), 107.0125 (3.48)	6.08	623.1606	−0.703	isorhamnetin-3-*O*-rutinoside ^1,2,3,4,5,6^	*
**Sesquiterpenes**								
**58**	229.1220	C_15_H_17_O_2_	229.1220 (100), 211.1116 (12.69), 201.1272 (19.57), 183.1167 (40.99), 91.0548 (6.40),	6.10	229.1223	−3.643	chamazulene carboxylic acid ^1,2,3^	[25]
**59**	245.1170	C_15_H_17_O_3_	245.1170 (100), 227.1067 (44.35), 217.1222 (20.20), 201.0910 (20.68), 199.1116 (75.08), 185.0959 (28.15), 95.0497 (66.93)	10.48	245.1172	−0.942	dehydroleucodin/isodehydroleucodin ^1,2,3^	[26]
**60**	245.1171	C_15_H_17_O_3_	245.1171 (84.77), 227.1065 (44.23), 217.1225 (17.55), 201.0910 (17.75), 199.1116 (63.86), 185.0960 (18.51), 95.0497 (100)	10.48	245.1172	−0.942	dehydroleucodin/isodehydroleucodin ^1,2,3^	[26]
**61**	247.1325	C_15_H_19_O_3_	247.1325 (100), 229.1220 (90.72), 211.1113 (8.83), 201.1271 (37.99), 187.0752 (37.99), 183.1167 (22.85), 91.0548 (9.16)	9.79	247.1328	−1.663	desacetoxymatricarin ^1,2,3,4,5,6^	**
**62**	249.1481	C_15_H_21_O_3_	249.1481 (100), 231.1378 (30.41), 213.1277 (9.04), 203.1431 (10.25), 193.0856 (5.87), 189.1272 (11.79), 159.1167 (22.04), 119.0857 (16.94), 105.0702 (18.62), 95.0860 (10.61)	10.42	249.1485	−0.451	parthenolide ^1,2,3,4,5,6^	**
**63**	263.1275	C_15_H_19_O_4_	263.1275 (100), 245.1170 (55.39), 227.1061 (9.88), 219.1021 (15.59), 217.1223 (47.56), 203.1065 (10.39), 199.1115 (21.45), 191.0704 (28.72), 95.0497 (96.99)	10.52	263.1277	−0.316	hydroxyleucodin ^1,2,3^	**
**64**	263.1274	C_15_H_19_O_4_	263.1274 (100), 245.1171 (33.08), 227.1063 (4.95), 219.1012 (14.30), 217.1224 (16.65), 203.1065 (10.12), 199.1117 (15.19), 191.0702 (79.26), 95.0497 (20.99)	11.22	263.1277	−0.406	hydroxyleucodin isomer ^1,2,3^	**
**65**	263.1275	C_15_H_19_O_4_	263.1275(100), 245.1170 (51.98), 227.1063 (11.55), 219.1014 (34.60), 217.1223 (47.24), 203.1067 (12.57), 199.1117 (22.59), 191.0702 (32.46), 95.0497 (81.14)	11.22	263.1277	−0.406	hydroxyleucodin isomer ^1,2,3^	**
**66**	265.1432	C_15_H_21_O_4_	265.1432 (8.16), 247.1326 (34.45), 229.1221 (100), 211.1115 (3.94), 201.1273 (20.45), 187.0752 (14.30), 183.1168 (7.67), 91.0548 (5.66)	6.04	265.1434	−0.813	stizolin ^1,3^	**
**67**	265.1429	C_15_H_21_O_4_	265.1429 (24.21), 247.1325 (100), 229.1222 (71.63), 219.1372 (36.25), 201.1272 (55.14), 187.1111 (17.50), 183.1174 (17.90), 91.0548 (13.40)	9.78	265.1434	−1.982	stizolin isomer ^1,3^	**
**68**	305.1360	C_17_H_21_O_5_	305.1360 (44.97), 287.1268 (3.27), 269.1206 (1.63), 263.1274 (42.58), 245.1170 (100), 227.1065 (63.42), 217.1220 (60.59), 109.1116 (23.36), 201.0903 (12.29), 185.0958 (19.24), 181.1010 (44.17), 171.1166 (56.51), 105.0703 (50.85), 95.0496 (25.16)	7.05	305.1383	−7.538	matricarin ^3^	**
**69**	305.1363	C_17_H_21_O_5_	305.1363 (23.50), 263.1279 (20.85), 245.1170 (100), 227.1063 (29.92), 217.1216 (19.51), 201.0911 (2.62), 185.0961 (16.47), 181.1009 (28.17), 171.1167 (31.70), 131.0857 (46.50), 95.0496 (3.40)	5.42	305.1383	−6.654	matricarin isomer ^3^	**
**70**	307.1544	C_17_H_21_O_5_	307.1544 (2.15), 289.1433 (0.75), 267.2721 (0.18), 247.1325 (2.63), 229.1219 (100), 211.1115 (0.95), 183.1168 (2.88)	9.67	307.1540	1.366	ludalbin ^1,2,3^	**

^1^-*A. cretica* MeOH extract, ^2^-*A. cretica* aqueous extract, ^3^-*A. cretica* EA extract, ^4^-*A. palida* MeOH extract, ^5^-*A. palida* aqueous extract, ^6^-*A. palida* EA extract. *-comparison with standard substance. **-tentatively identification.

**Table 3:** Antioxidant properties of *Anthemis* extracts *.

Plant Names	Solvents	Phosphomolybdenum (mmol TE/g)	DPPH (mg TE/g)	ABTS (mg TE/g)	CUPRAC (mgTE/g)	FRAP (mgTE/g)	Metal Chelating Abilitiy (mg EDTAE/g)
*A. tinctoria* var. *pallida*	Ethyl acetate	2.59 ± 0.19 ^b^	40.30 ± 0.78 ^e^	45.52 ± 5.53 ^f^	113.31 ± 2.26 ^d^	47.63 ± 3.77 ^f^	39.01 ± 4.42 ^a^
	Methanol	2.99 ± 0.14 ^a^	407.07 ± 8.88 ^a^	320.11 ± 5.67 ^a^	691.17 ± 12.07 ^a^	362.12 ± 2.63 ^a^	28.28 ± 1.81 ^c^
	Aqueous	2.65 ± 0.02 ^b^	298.40 ± 6.74 ^b^	303.16 ± 8.57 ^b^	584.01 ± 8.71 ^b^	316.34 ± 4.15 ^b^	33.59 ± .16 ^b^
*A. cretica* subsp. *tenuiloba*	Ethyl acetate	1.69 ± 0.08 ^cd^	45.47 ± 2.16 ^e^	57.13 ± 3.89 ^e^	112.87 ± 4.41 ^d^	55.74 ± 2.27 ^e^	21.90 ± 0.81 ^d^
	Methanol	1.77 ± 0.08 ^c^	97.22 ± 0.22 ^c^	112.41 ± 2.35 ^d^	223.09 ± 6.17 ^c^	143.21 ± 1.77 ^d^	20.93 ± 1.70 ^d^
	Aqueous	1.56 ± 0.05 ^d^	86.74 ± 2.46 ^d^	127.68 ± 0.45 ^c^	214.45 ± 1.39 ^c^	130.86 ± 1.81 ^c^	39.64 ± 1.34 ^a^

* Values expressed are means ± S.D. of three parallel measurements. TE: Trolox equivalent; EDTAE: EDTA equivalent. Different letters indicate significant differences in the extracts (*p* < 0.05).

**Table 4:** Enzyme inhibitory activity of *Anthemis* extracts *.

Plant Names	Solvents	AChE Inhibition (mg GALAE/g)	BChE Inhibition (mg GALAE/g)	Tyrosinase Inhibition (mg KAE/g)	Amylase Inhibition (mmol ACAE/g)	Glucosidase Inhibition (mmol ACAE/g)
*A. tinctoria* var. *pallida*	Ethyl acetate	1.33 ± 0.03 ^c^	3.48 ± 0.21 ^a^	124.60 ± 0.15 ^b^	0.78 ± 0.05 ^a^	21.94 ± 1.91 ^b^
	Methanol	3.28 ± 0.43 ^b^	na	124.48 ± 1.23 ^b^	0.54 ± 0.02 ^c^	9.15 ± 2.26 ^c^
	Aqueous	na	na	72.10 ± 1.64 ^d^	0.11 ± 0.01 ^d^	4.95 ± 0.25 ^d^
*A. cretica* subsp. *tenuiloba*	Ethyl acetate	4.68 ± 0.21 ^a^	2.51 ± 0.34 ^b^	128.73 ± 0.71 ^a^	0.65 ± 0.01 ^b^	24.16 ± 0.12 ^a^
	Methanol	3.45 ± 0.26 ^b^	1.15 ± 0.05 ^c^	128.85 ± 1.41 ^a^	0.52 ± 0.06 ^c^	4.49 ± 0.93 ^d^
	Aqueous	na	na	88.95 ± 0.49 ^c^	0.09 ± 0.01 ^d^	2.49 ± 0.17 ^e^

* Values expressed are means ± S.D. of three parallel measurements. GALAE: Galatamine equivalent; KAE: Kojic acid equivalent; ACAE: Acarbose equivalent; na: not active. Different letters indicate significant differences in the extracts (*p* < 0.05).

## Supplementary Materials

The following are available online. Supplementary Data 1; Supplementary Data 2; Supplementary Proteomic Analysis.
